# The Relationships Between Short Video Usage and Subjective Well-Being: Mediation Models and Network Analysis

**DOI:** 10.3390/bs14111082

**Published:** 2024-11-12

**Authors:** Guanghui Zhai, Jiahui Su, Zhouying Chen, Yang Feng, Yunpeng Jiang, Tour Liu, Xia Wu

**Affiliations:** 1 Faculty of Psychology, Tianjin Normal University, Tianjin 300387, China; 2210340006@stu.tjnu.edu.cn (G.Z.); ssucog@gmail.com (J.S.); 2211340153@stu.tjnu.edu.cn (Z.C.); psyfy1997@163.com (Y.F.); jiangyp@tjnu.edu.cn (Y.J.); mikebonita@hotmail.com (T.L.); 2 Key Research Base of Humanities and Social Sciences of the Ministry of Education, Academy of Psychology and Behavior, Tianjin 300387, China; 3 Tianjin Social Science Laboratory of Students’ Mental Development and Learning, Tianjin 300387, China

**Keywords:** short video, active usage, passive usage, subjective well-being, social anxiety, network analysis

## Abstract

On short video platforms, individuals can not only passively browse videos but also actively engage in behaviors such as liking and commenting on videos. However, the mechanisms by how active and passive usage of short videos affect subjective well-being remains unclear. Thus, this study aims to explore these mechanisms through path analysis and network analysis. We employed the Short Video Usage Questionnaire, Subjective Well-Being Scale, and Interaction Anxiety Scale to survey 1086 participants. The results indicated (1) the active usage positively predicted life satisfaction and positive affect, while passive usage failed to predict any of them. (2) Social anxiety mediated the relationship between short video usage and subjective well-being. Active usage enhanced life satisfaction and positive affect by alleviating social anxiety, while reducing negative affect by decreasing social anxiety. At the same time, passive usage reduced life satisfaction and positive affect by exacerbating social anxiety while increasing negative affect by intensifying social anxiety. (3) Network analysis revealed that “live interaction” behavior was a core activity for alleviating social anxiety and enhancing subjective well-being. The findings highlight the significant role of short video usage in subjective well-being and provide empirical evidence to guide users in the rational and positive usage of short video platforms.

## 1. Introduction

Short videos feature brief clips ranging from 15 s to a few minutes in length [1]. This emergent form of social media has rapidly become popular. As short videos increasingly permeate daily life, their impact on individual psychological health manifest both positively and negatively. Positively, they serve to provide entertainment [1], foster and strengthen social connections [2], and improve subjective well-being [3]. However, short videos can also lead to some psychological issues such as anxiety [4] and addictive behaviors [5].

The mode of short video usage, besides the duration and intensity of usage, may be an important variable that influences psychological health [6]. Social media usage can be categorized into active and passive forms [7]. As a type of social media, short video usage can also be classified in this way. Active usage involves interactions that facilitate direct exchanges with others, such as commenting on videos or engaging in discussions during live streams. While passive usage refers to monitoring other people’s lives without engaging in direct exchanges, such as viewing others’ videos and comments. Studies indicated that users who frequently commented on social networks can expand their social connections [2]. By contrast, passive viewers face higher risks of loneliness [8] and envy [9]. Therefore, investigating the different roles of active and passive short video usage on psychological health not only helps to provide a comprehensive view of the impact of short videos but also offers insights for personalized guidance, enhancing social and entertainment benefits while minimizing potential adverse effects.

Active and passive short video usages may have various effects on subjective well-being. Subjective well-being is a reflection of an individual’s quality of social life [10]. Verduyn developed an extended active–passive usage model to provide a more precise explanation of the relationship between social media usage patterns and subjective well-being [11]. In the model, active usage can accumulate social capital (i.e., social support) while fulfilling the need for interpersonal relationships, ultimately enhancing well-being [12,13]. In contrast, passive usage can harm subjective well-being through upward social comparison related to self-concept [14]. However, there are two aspects where the current research can be improved. First, compared to Facebook, short video platforms rely on personalized recommendation algorithms to deliver content. Thus, when using short video, the connections between users are based on content similarity rather than actual social relationships, meaning that social capital between users are weaker and further removed from their self-concept. Secondly, subjective well-being can be divided into affective components (i.e., positive affect and negative affect) and cognitive components (i.e., life satisfaction) [15]. Some researchers have only found the impact of usage patterns on the affective component [16,17], while others have identified effects solely on the cognitive component [3]. Therefore, this study aimed to investigate the effects of usage patterns on each subcomponent of subjective well-being within the context of short video platforms. We hypothesized that active usage can increase life satisfaction and positive affect while decreasing negative affect (H_1_). Conversely, passive usage can increase negative affect but decrease both life satisfaction and positive affect (H_2_).

Active and passive short video usage could affect social anxiety, which in turn could negatively impact subjective well-being. Thus, social anxiety may serve as a mediator while short video usage influencing subjective well-being. Social anxiety is defined as individual’s irrational fear of being scrutinized or judged in social situations [18]. When an individual experiences social anxiety, it triggers a series of psychophysiological responses, including rapid breathing, facial flushing, and increased cortisol secretion, and may also lead to internal discomfort, such as gastrointestinal disturbances [19]. Such individuals often adopt passive coping mechanisms, including silence and avoidance, to escape the tension and discomfort of social situations [20]. Firstly, the short video usage directly influences social anxiety. In the virtual world, social pressure is slighter relative to reality [21]. In the virtual world, users experience less social pressure compared to reality [21], which leads to a lower physiological stress response [22]. They are more willing to actively share opinions and seek social support through likes and comments. This process can enhance social skills, raise self-esteem, and reduce social anxiety [7]. In contrast, passive usage involves observing the external world without interactive communication, which does not actively build interpersonal relationships, potentially increasing levels of social anxiety [23]. Secondly, social anxiety can reduce subjective well-being. Previous studies have found that individuals with high social anxiety experience lower subjective well-being [24,25]. This is because their tension and fear increase negative affect and reduce positive affect, while irrational self-beliefs also decrease satisfaction with one’s life [24]. Consequently, we hypothesized that social anxiety mediated the relationship between usage and subjective well-being. Specifically, active usage can decrease social anxiety, thereby enhancing life satisfaction and positive affect, while reducing negative affect (H_3_). Meanwhile, passive usage can increase social anxiety, thereby increasing negative affect, while reducing life satisfaction and positive affect (H_4_).

Different short video usage behaviors may have varying psychological impacts. For example, research on Facebook has shown that browsing messages increased feelings of loneliness [8], whereas posting messages can promoted self-disclosure [26]. It is meaningful to explore the role of specific behaviors of short video usage on psychological variables in this study. Besides path analysis, we incorporated network analysis as well. Network analysis can incorporate specific behaviors and psychological variables as nodes within a network, identify core factors through relevant statistical metrics, and graphically display the relationships among them [27]. The aim of path analysis was to validate the relationships between variables based on a priori models, while the network analysis, as an exploratory technique, sought to identify the key variables affecting the model. As a result, both approaches were complementary and the combined validation could ensure greater robustness.

Therefore, we aimed to investigate the impact of different short video usage behaviors on subjective well-being and the mediating role of social anxiety through path analysis. Subsequently, we utilized network analysis to identify the specific usage behavior with the greatest contribution to the model. This study provides a theoretical basis for alleviating social anxiety and improving well-being, as well as practical implications for designing short video platforms that are more beneficial to mental health.

## 2. Methods

### 2.1. Procedure and Participants

The participants of this study were drawn from various provinces in China. Data collection was conducted in March 2023 using an online platform, yielding a total of 1427 responses. After removing participants who failed two lie detection questions, had excessively fast response times, and reported excessively long short video usage times, 1086 valid responses were retained. The lie detection questions include “When was the People’s Republic of China established?” and “Please select ‘Yes’ for this question. “The sample comprised 465 males (42.8%) and 622 females (57.2%), with an average age of 20.95 ± 1.94 years. In addition, 87.2% of the participants had an educational background of undergraduate or lower levels, while 12.8% had more than an undergraduate level. A total of 54.3% of the participants lived in rural areas, while 45.7% lived in urban areas. 40.2% of the participants were only children, and 59.8% were non-only children. The study was approved by the ethical committee of Tianjin Normal University. All participants provided consent for using their data in the study.

### 2.2. Measures

Short Video Usage: the Short Video Usage Questionnaire developed by Li was adapted to fit the context of short video usage [28]. Participants responded to the items on a 6-point scale ranging from “1 = never” to “6 = several times a day” to assess the frequency of each short video usage behavior. Additionally, three items characteristic of short videos were added: “I save videos on the short video App”, “I participate in interactions such as communicating with others or sending gifts during live broadcasts on the short video App”, and “I search for videos I want to watch on the short video App”. The scale originally comprised 10 items. After exploratory and confirmatory factor analyses, three items were removed. The passive dimension retained two items, namely “browse videos” and “read comments”. The active dimension retained five items, namely “like”, “save”, “comment”, “share”, and “live interaction”. Confirmatory factor analysis yielded the following results: χ2df = 5.177, TLI = 0.914, CFI = 0.947, GFI = 0.966, RMSEA = 0.088. Cronbach’s alpha was 0.752 for the active usage subscale (5 items) and 0.721 for the passive usage subscale (2 items).

Social Anxiety: the Interaction Anxiety Scale (IAS) developed by Leary [29] was later translated into Chinese by Wang [30]. Participants were asked to respond on a five-point Likert scale from “1 = strongly disagree” to “5 = strongly agree”. It contained 15 items to evaluate level of social anxiety. Cronbach’s alpha for this scale was 0.915.

Subjective Well-Being: the measurement of subjective well-being included two components: life satisfaction and the assessment of positive and negative affect [15]. The life satisfaction scale was originally developed by Diener in 1985 [31]. This study used the life satisfaction scale (LSS), revised by Zhang [32]. It consisted of 5 items scored on a 7-point scale, where 1 is “strongly disagree” and 7 is “strongly agree”. A higher score indicated greater satisfaction with one’s current life circumstances, with a Cronbach’s alpha of 0.891. The measurement of positive and negative affects used the Positive Affect and Negative Affect Scale (PANAS), developed by Watson [33] and revised by Qiu [34]. It was divided into two dimensions: positive and negative affect, each with 9 items scored on a 5-point scale, where 1 is “none or very slight” and 5 is “very strong”. A higher score indicated stronger corresponding emotions over the past week. Cronbach’s alpha for the positive affect subscale was 0.898, and for the negative affect subscale, it is 0.906. When calculating the total subjective well-being score (Cronbach α = 0.930), negative affect was reverse scored and combined with positive affect and life satisfaction.

Covariates included age (ranging from 18 to 28), gender (1 = male and 0 = female), and average daily usage time of short videos (hours).

### 2.3. Analysis

#### 2.3.1. Descriptive Statistics and Mediation Testing

IBM SPSS 27.0.1.0 was used for the data analysis. Path modeling was performed using the SPSS macro PROCESS (version 4.0). AMOS 28.0 was used for confirmatory factor analysis. The correlation analysis was conducted by corrplot package (version 0.92) for R (version 4.3.2).

#### 2.3.2. Network Analysis

Network analysis can be used to analyze correlation or partial correlation relationships between variables in quantitative and graphical ways. By means of the graph theory, networks depicted the components of a system (i.e., nodes) and the links between these components (i.e., edges). Nodes in the same cluster are of the same color. Green edges indicated positive correlations, while red edges indicated negative correlations. Thicker and darker edges signified stronger associations between nodes. Partial correlation was used to control for spurious correlations.

The Graphical Least Absolute Shrinkage and Selection Operator (GLASSO) model, combined with the Extended Bayesian Information Criterion (EBIC), was utilized for model selection to obtain a more stable and interpretable regularized partial correlation network. GLASSO was a regularization technique that reduced small correlations to zero by assigning penalties, thus limiting the number of false positives and creating a more interpretable and sparse network [35]. EBIC, a model selection fit index, allowed for control over model sparsity through the use of the hyperparameter gamma [36].

Centrality indices represented a node’s relationship with other nodes in the network, with the most used commonly being strength centrality, closeness centrality, and betweenness centrality. Strength centrality was the sum of the weights of the edges connected to a node, reflecting the direct connection of the node with other nodes. Closeness centrality was the reciprocal of the sum of the shortest distances to all other nodes, examining the node’s indirect connections with others. Betweenness centrality referred to the proportion of all shortest paths that pass through a node, highlighting its importance in connecting other nodes [37]. However, for psychological networks, betweenness and closeness centrality seemed especially unsuitable as measures of node importance [38]. Therefore, this study placed greater emphasis on strength centrality.

Edge stability was estimated by 95% confidence intervals obtained by bootstrapping, with smaller overlaps between intervals indicating higher stability. The stability of node centrality estimates was assessed with the centrality stability coefficient (CS coefficient), where a coefficient greater than 0.25 indicated acceptable stability and values above 0.5 denoted good stability.

Network analysis was conducted using R (version 4.3.2). The qgraph package (version 1.9.8) was used to estimate and visualized the partial correlation network of the sample [39]. The bootnet package (version 1.5.6) was used to test the accuracy of the edges and the stability of node centrality in the network [35].

### 2.4. Common Method Bias Test

In terms of procedure, this study adopted measures such as anonymous survey completion, uniform instructions, and standardized testing processes to reduce common method bias [40]. Statistically, the Harman single-factor test was employed to assess common method bias. It was found that there were 10 factors with eigenvalues greater than 1, and the first common factor explained only 24.41% of the total variance, which is below the 40% threshold. To enhance the reliability of the results further, confirmatory factor analysis was conducted, setting the common factor at 1 to verify model fit. The results indicated poor model fit (χ2df = 17.49, RMSEA = 0.12, CFI = 0.45, TLI = 0.42). Therefore, there was no interference from common method bias in this study.

## 3. Results

### 3.1. Descriptive Statistics and Correlation Analysis

**Figure 1:**
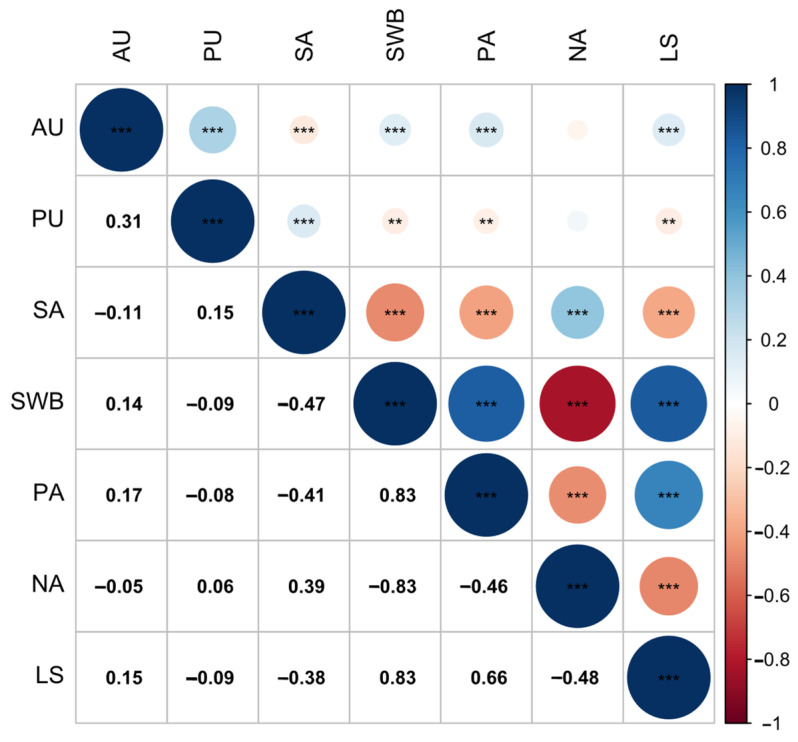
Correlations and Significance of Various Variable Dimensions. AU, active usage; PU, passive usage; SA, social anxiety; SWB, subjective well-being; PA, positive affect; NA, negative affect; LS, life satisfaction. The bottom left shows the correlation coefficients, while the top right shows the significance of the corresponding correlation coefficients. The areas of circles or squares show the absolute value of the corresponding correlation coefficients. The color represents the direction of the correlation. The number of asterisks indicates the level of significance (***: *p* < 0.001, **: *p* < 0.01).

The correlation analysis for each variable is presented in Figure 1. The results indicated that active usage was significantly positively correlated with subjective well-being, positive affect, and life satisfaction, and was significantly negatively correlated with social anxiety. Passive usage was significantly positively correlated with social anxiety and significantly negatively correlated with subjective well-being, positive affect, and life satisfaction.

### 3.2. Test for Mediation Models

#### 3.2.1. Test for Mediation Models of Active Usage

**Figure 2:**
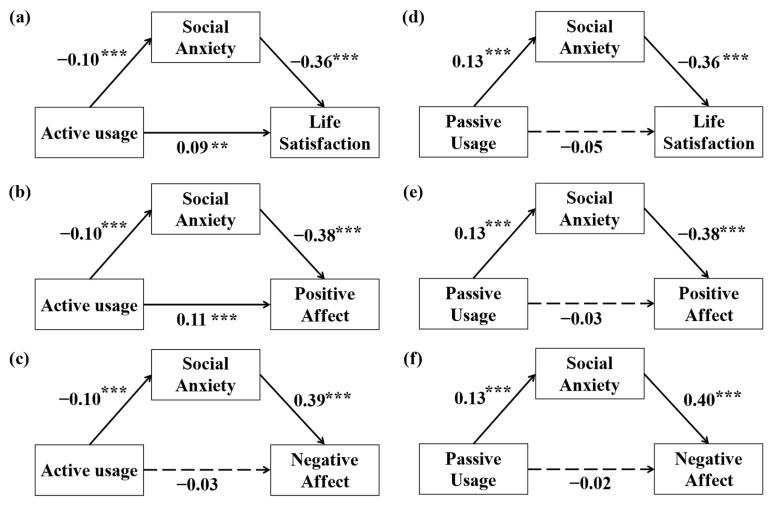
Subfigures (**a**–**c**) are path diagrams with active usage as the independent variable, social anxiety as the mediator, and life satisfaction, positive affect, and negative affect as the dependent variables, respectively. Subfigures (**d**–**f**) are path diagrams with passive usage as the independent variable, social anxiety as the mediator, and life satisfaction, positive affect, and negative affect as the dependent variables, respectively. Further model details can be found in the Supplementary Information. Note: ***: *p* < 0.001, **: *p* < 0.01.

The mediation effect of social anxiety between active usage and life satisfaction was tested using Model 4 of the PROCESS written by Hayes [41]. Covariates included the average daily usage time of short videos (ADUT), gender, and age. The same is applied below. The path diagram of active usage to life satisfaction was presented in Figure 2a. The direct predictive effect of active usage on life satisfaction was significant (*β* = 0.13, *t* = 4.11, *p* < 0.001). When the mediating variable of social anxiety was included, this direct effect remained significant (*β* = 0.09, *t* = 3.12, *p* = 0.002). The negative predictive effect of active usage on social anxiety was significant (*β* = −0.10, *t* = −3.37, *p* = 0.001), as was the negative predictive effect of social anxiety on life satisfaction (*β* = −0.36, *t* = −12.12, *p* < 0.001). Social anxiety had a significant mediation effect on the relationship between active usage and life satisfaction (*β* = 0.04, SE = 0.01, 95% CI = [0.016, 0.061]), as the confidence interval did not include zero.

The path diagram of active usage to positive affect was presented in Figure 2b. The direct predictive effect of active usage on positive affect was significant (*β* = 0.15, *t* = 4.84, *p* < 0.001). When the mediating variable of social anxiety was included, this direct effect remained significant (*β* = 0.11, *t* = 3.84, *p* < 0.001). The negative predictive effect of active usage on social anxiety was significant (*β* = −0.10, *t* = −3.37, *p* = 0.001), as was the negative predictive effect of social anxiety on positive affect (*β* = −0.38, *t* = −13.10, *p* < 0.001). Social anxiety had a significant mediation effect on the relationship between active usage and positive affect (*β* = 0.04, SE = 0.01, 95% CI = [0.015, 0.064]), as the confidence interval did not include zero.

The path diagram of active usage to negative affect was presented in Figure 2c. The direct predictive effect of active usage on negative affect was not significant (*β* = −0.07, *t* = −2.32, *p* = 0.027). When the mediating variable of social anxiety was included, this direct effect was not significant (*β* = −0.03, *t* = −1.02, *p* = 0.308). The negative predictive effect of active usage on social anxiety was significant (*β* = −0.10, *t* = −3.37, *p* = 0.001), as was the positive predictive effect of social anxiety on negative affect (*β* = 0.39, *t* = 13.31, *p* < 0.001). Social anxiety had a significant mediation effect on the relationship between active usage and negative affect (*β* = −0.04, SE = 0.01, 95% CI = [−0.066, −0.017]), as the confidence interval did not include zero.

#### 3.2.2. Test for Mediation Models of Passive Usage

Employing Model 4 from the PROCESS written by Hayes [41], the mediation effect of social anxiety in the relationship between passive usage and life satisfaction was tested. The path diagram of passive usage to life satisfaction was presented in Figure 2d. Passive usage had a significant direct effect on life satisfaction (*β* = −0.10, *t* = −3.14, *p* = 0.002). However, when the mediating variable of social anxiety was included, this direct effect became non-significant (*β* = −0.05, *t* = −1.70, *p* = 0.09), suggesting that social anxiety fully mediated the path from passive usage to life satisfaction. The positive predictive effect of passive usage on social anxiety was significant (*β* = 0.13, *t* = 4.42, *p* < 0.001), as is the negative predictive effect of social anxiety on life satisfaction (*β* = −0.36, *t* = −12.13, *p* < 0.001). Social anxiety had a significant mediation effect on the relationship between passive usage and life satisfaction (*β* = −0.05, SE = 0.01, 95% CI = [−0.071, −0.025]), as the confidence interval did not include zero.

The path diagram of passive usage to positive affect was presented in Figure 2e. Passive usage had a significant direct effect on positive affect (*β* = −0.08, *t* = −2.68, *p* = 0.008). However, when the mediating variable of social anxiety was included, this direct effect became non-significant (*β* = −0.03, *t* = −1.08, *p* = 0.281), suggesting that social anxiety fully mediated the path from passive usage to positive affect. The positive predictive effect of passive usage on social anxiety was significant (*β* = 0.13, *t* = 4.42, *p* < 0.001), as is the negative predictive effect of social anxiety on positive affect (*β* = −0.38, *t* = −13.21, *p* < 0.001). Social anxiety had a significant mediation effect on the relationship between passive usage and positive affect (*β* = −0.05, *SE* = 0.01, 95% CI = [−0.077, −0.028]), as the confidence interval did not include zero.

The path diagram of passive usage to negative affect was presented in Figure 2f. Passive usage did not have a significant direct effect on negative affect (*β* = 0.04, *t* = 1.20, *p* = 0.233). When the mediating variable of social anxiety was included, this direct effect was also non-significant (*β* = −0.02, *t* = −0.51, *p* = 0.609). The positive predictive effect of passive usage on social anxiety was significant (*β* = 0.13, *t* = 4.42, *p* < 0.001), as is the positive predictive effect of social anxiety on negative affect (*β* = 0.40, *t* = 13.43, *p* < 0.001). Social anxiety had a significant mediation effect on the relationship between passive usage and negative affect (*β* = 0.05, SE = 0.01, 95% CI = [0.028, 0.077]), as the confidence interval did not include zero.

### 3.3. Network Analysis Results

**Figure 3:**
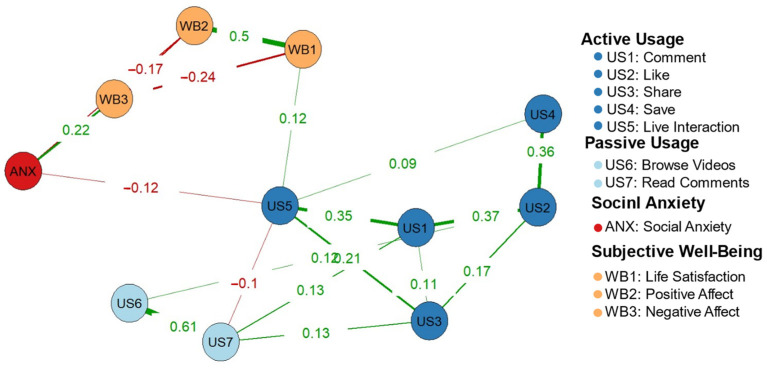
Network diagram of short video usage, social anxiety, and subjective well-being.

**Figure 4:**
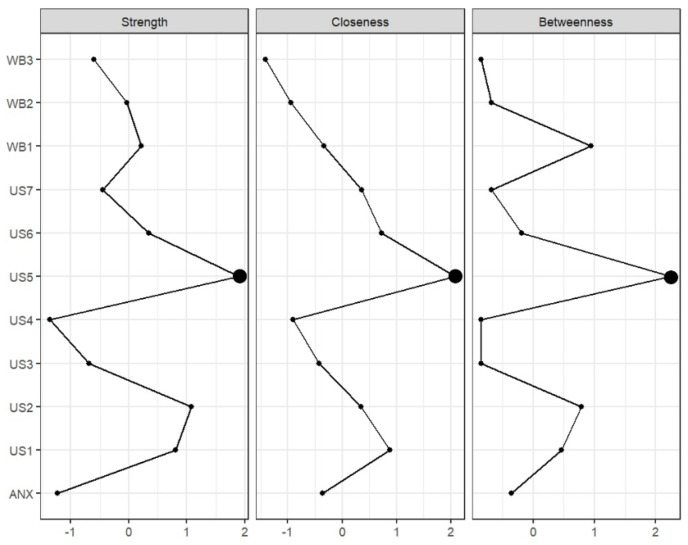
Centrality indices of the short video usage network.

The regularized partial correlation network for active usage is shown in Figure 3. The diagram indicated that “live interaction” was negatively related to social anxiety and positively related to “life satisfaction” within subjective well-being. The accuracy of the edges, indicated by confidence intervals that overlap less for stronger connections, suggested that the overall network estimate was fairly accurate. The three types of centrality indices are displayed in Figure 4, with centrality stability coefficients (CS) as follows: strength = 0.595, closeness = 0.439, and betweenness = 0.361. The CS coefficient greater than 0.25 indicated acceptable stability, and values above 0.5 denoted good stability [35]. These findings suggested that the centrality estimates in the network were stable, and the results were highly interpretable.

## 4. Discussion

Combining mediation models and network analysis, the principal findings of the present study were that active usage directly or indirectly predicted the three subcomponents of subjective well-being. However, passive usage could only indirectly predict them through social anxiety. Network analysis pinpointed “live interaction” as the node with the highest strength centrality. This study extends our understanding of the effects of short videos, the most widely used digital media in our daily lives, on subjective well-being. Moreover, it provides guidance for users on the rational use of short videos in the future.

Active and passive usage of short videos have different impacts on subjective well-being. Subjective well-being can be divided into three dimensions: positive affect, negative affect, and life satisfaction [10]. Consistent with prior studies on other social media platforms, active usage positively predicted life satisfaction [6] and positive affect [16]. During the active usage of short videos, individuals express their support for others’ opinions by liking, saving, and sharing and exchange information by commenting and live interactions [42]. Besides providing support, active users may also have the opportunity to receive support from others [43]. According to social support theory, when feeling valued and cared for, people can experience greater health and well-being [44]. However, contrary to previous studies [16], active usage did not alleviate negative affect. It is possible that when individuals feel sad and turn to friends for comfort, they are more likely to use Facebook rather than TikTok [45]. For passive usage, it only indirectly predicted subjective well-being. During the passive usage of short videos, users have the freedom to browse a wide range of contents without any restrictions. Different contents have been proven to have various impacts on the well-being [46]. For example, individuals who view people/fashion-themed videos tend to have lower life satisfaction compared to those who watch entertainment/relaxation-themed videos [3]. Besides content, upward social comparison or envy has also been proven to fully mediate the path from passive usage of social media to subjective well-being [17]. Therefore, without considering the above factors, passive usage may not have a direct impact on subjective well-being. By distinguishing usage types of short videos, the present study extends the impact of social media on our psychological well-being and finds different impacts on it.

Moreover, social anxiety mediated the path of the effects of short video usage on subjective well-being. Active usage can improve subjective well-being by reducing social anxiety, while passive usage can decrease subjective well-being by increasing social anxiety. During the active usage of short videos, communication with others on the website can reduce stress and anxiety compared to real-world life [21]. In such a relaxed environment, individuals are more willing to express their opinions and less likely to experience social anxiety. Furthermore, the personalized recommendation algorithms of short videos can facilitate interactions between individuals with similar interests [47]. The users can more easily obtain positive feedback and refrain themselves from social anxiety [42,48]. Previous studies have found that high-quality communication in virtual environments can improve physiological indicators of anxiety, such as electrodermal activity and cortisol levels [22,49]. contrary to active usage, passive usage can only indirectly predict subjective well-being through social anxiety. Passive users do not engage in social interactions and lack the opportunity to obtain social support. Additionally, the beautified information can lead to upward social comparisons and envy, resulting in negative self-evaluations and thereby triggering social anxiety [14]. For the path from social anxiety to subjective well-being, we found that social anxiety was negatively associated with life satisfaction and positive affect, while positively associated with negative affect. Because the tension and unease associated with social anxiety can impair an individual’s emotional experience (i.e., the affective component of subjective well-being), and the irrational beliefs of self can lower their cognitive evaluations of life (i.e., the cognitive component of subjective well-being), thus harming subjective well-being [24]. This study examined the impact of short video usage on subjective well-being with social anxiety serving as a mediating variable. It offers a new theoretical perspective and explores how media usage in the virtual world can change real-world social anxiety and, consequently, subjective well-being.

Through network analysis, this study identified that “live interaction” of active usage may be a key factor influencing social anxiety and subjective well-being. First, “live interaction” was negatively correlated with social anxiety. Compared to other active usage behaviors (e.g., commenting), “live interaction” more closely resembles real-world social behavior, which requires real-time understanding and feedback. In addition, “live interaction” involves less social pressure than real-world social interactions, allowing individuals to receive encouraging feedback within groups of similar interests, which further reduces social anxiety. Second, “live interaction” had a positive association with the “life satisfaction” of subjective well-being. As a cognitive aspect of subjective well-being, life satisfaction is termed as an overall personal cognitive assessment of one’s life condition for a substantial period [10]. Life satisfaction is closely linked to good interpersonal relationships and a sense of belonging [50]. Personalized recommendation algorithms allow individuals to interact live with others who share similar interests. These interactions can facilitate emotional resonance and value affirmation, thus enhancing individuals’ sense of social belonging. We can also use the extended active–passive usage model to explain this result [46]. According to this theory, active usage enhances subjective well-being through the increase in social capital (i.e., social support), and this increase is constrained by reciprocity and communion. When the host or viewers engage in a discussion on a particular topic, the exchange of opinions and information can enhance the accumulation of social capital. Compared to non-real-time behaviors like liking and commenting, the online live interactions are more likely to expect a response, leading to higher reciprocity. Additionally, since online communication is often positive, it tends to be warm [51]. Therefore, this behavior accumulates more social capital compared to other forms of active usage. Similar to this study, previous studies found that another active usage behavior, “posting short videos” can also predict a higher level of life satisfaction [3]. Previous research has explored the impact of common usage behaviors like commenting [52] and posting [3] on psychological variables. In this study, the significant influence of “live interaction” has been found by using network analysis to visually illustrate the relationships between variables. Future research can more specifically investigate live interaction behaviors such as giving gifts.

In summary, this study investigated the impact mechanism of short video usage on subjective well-being through path analysis and identified the central role of live interaction in the model through network analysis. The outcomes of this research offer numerous potential applications. For instance, live interactions on short video platforms could be employed as a supportive tool in therapy. The core of therapy for social anxiety is exposure, which involves confronting feared stimuli while eliminating safety behaviors, allowing patients to understand that the negative consequences they fear are unlikely to occur [53]. Recent research has found that Virtual Reality Exposure Therapy, which gradually exposes patients to simulated social environments through virtual reality technology, can effectively alleviate symptoms [22,49]. Live interactions may serve as a supplementary component of this therapy in everyday life. Furthermore, drawing from Human–Computer Interaction (HCI) theory, it is advisable for platform designers to introduce features such as anonymous commenting or silent engagement modes, which allow users to engage incrementally without disclosing personal identities, ultimately fostering a greater sense of well-being [54]. These features are likely to enhance user comfort and participation, particularly for individuals who experience anxiety or vulnerability in social interactions.

This study has several limitations. The cross-sectional design limits the ability to draw causal inferences. Therefore, longitudinal studies are necessary to establish causality and examine the temporal relationships among short video usage patterns, social anxiety, and subjective well-being. Furthermore, self-reported data may lead to various issues, such as self-bias, memory errors, comprehension differences, social desirability effects, and the influence of emotional states. Future research can utilize a combination of various objective measurement data, such as heart rate, skin conductance, cortisol levels, and structural and functional measurements from magnetic resonance imaging, to provide a more comprehensive understanding of their impact on mental health.

## Data Availability

Data are available at https://osf.io/e2xsr/ (accessed on 11 May 2023).

## Supplementary Materials

The following supporting information can be downloaded at: https://www.mdpi.com/article/10.3390/bs14111082/s1, Table S1: Testing the Mediation Model of Active Usage and Life Satisfaction. Table S2: Testing the Mediation Model of Active Usage and Positive Affect. Table S3: Testing the Mediation Model of Active Usage and Negative Affect. Table S4: Testing the Mediation Model of Passive Usage and Life Satisfaction. Table S5: Testing the Mediation Model of Passive Usage and Positive Affect. Table S6: Testing the Mediation Model of Passive Usage and Negative Affect.
