# Supplementary material for: The Relationships Between Short Video Usage and Subjective Well-Being: Mediation Models and Network Analysis
Source: Behav Sci (Basel). 2024 Nov 12;14(11):1082. doi: 10.3390/bs14111082 (PMC11590871; doi:10.3390/bs14111082)
Supplement: Supplementary file 1 [file behavsci-14-01082-s001.zip › behavsci-3287535-supplementary.pdf]

Table S1: Testing the Mediation Model of Active Usage and Life Satisfaction

Outcome Variable	Predictor Variable	R	R^2	F	β	t
Life Satisfaction		0.21	0.05	12.62***		
	ADUT				-9.02	-0.69
	gender				0.12	4.05***
	age				0.08	2.70**
	Active Usage				0.13	4.11***
Social Anxiety		0.30	0.09	26.83***		
	ADUT				0.11	3.66***
	gender				-0.21	-7.07***
	age				-0.14	-4.79***
	Active Usage				-0.10	-3.37***
Life Satisfaction		0.40	0.16	40.83***		
	ADUT				0.02	0.61
	gender				0.05	1.66
	age				0.03	1.10
	Active Usage				0.09	3.12**
	Social Anxiety				-0.36	-12.12***

Note: ADUT, the average daily usage time of short videos. All variables in the model have been standardized before being entered into the regression equation. The same applies below.

Table S2: Testing the Mediation Model of Active Usage and Positive Affect

Outcome Variable	Predictor Variable	R	R^2	F	β	t
Positive Affect		0.24	0.06	15.83***		
	ADUT				-9.02	-1.51
	gender				0.12	4.75***
	age				0.08	2.23*
	Active Usage				0.15	4.84***
Social Anxiety		0.30	0.09	26.83***		
	ADUT				0.11	3.66***
	gender				-0.21	-7.07***
	age				-0.14	-4.79***
	Active Usage				-0.10	-3.37***
Positive Affect		0.43	0.19	48.97***		
	ADUT				-0.01	-0.17
	gender				0.07	2.25*
	age				0.01	0.49
	Active Usage				0.11	3.84***
	Social Anxiety				-0.38	-13.10***

Table S3: Testing the Mediation Model of Active Usage and Negative Affect

Outcome Variable	Predictor Variable	R	R^2	F	β	t
Negative Affect		0.14	0.02	5.56***		
	ADUT				-0.07	-2.21*
	gender				0.10	3.21***
	age				-0.06	-1.80
	Active Usage				-0.07	-2.32*
Social Anxiety		0.30	0.09	26.83***		
	ADUT				0.11	3.66***
	gender				-0.21	-7.07***
	age				-0.14	-4.79***
	Active Usage				-0.10	-3.37***
Negative Affect		0.40	0.16	40.62***		
	ADUT				0.06	1.97*
	gender				0.03	0.90
	age				-0.02	-0.55
	Active Usage				-0.03	-1.02
	Social Anxiety				0.39	13.31***

Table S4: Testing the Mediation Model of Passive Usage and Life Satisfaction

Outcome Variable	Predictor Variable	R	R^2	F	β	t
Life Satisfaction		0.20	0.04	10.79***		
	ADUT				0.03	1.08
	gender				0.14	4.81***
	age				0.07	2.33*
	Passive Usage				-0.10	-3.14**
Social Anxiety		0.31	0.10	29.07***		
	ADUT				0.06	1.85
	gender				-0.23	-7.75***
	age				-0.13	-4.46***
	Passive Usage				0.13	4.42***
Life Satisfaction		0.39	0.15	39.20***		
	ADUT				0.05	1.82
	gender				0.06	2.21
	age				0.02	0.83
	Passive Usage				-0.05	-1.70
	Social Anxiety				-0.36	-12.13***

Table S5: Testing the Mediation Model of Passive Usage and Positive Affect

Outcome Variable	Predictor Variable	R	R^2	F	β	t
Positive Affect		0.20	0.04	11.60***		
	ADUT				0.01	0.34
	gender				0.17	5.67***
	age				0.05	1.82
	Passive Usage				-0.08	-2.68**
Social Anxiety		0.31	0.10	29.07***		
	ADUT				0.06	1.85
	gender				-0.23	-7.75***
	age				-0.13	-4.46***
	Passive Usage				0.13	4.42***
Positive Affect		0.42	0.18	45.68***		
	ADUT				0.03	1.11
	gender				0.08	2.91**
	age				0.01	0.16
	Passive Usage				-0.03	-1.08
	Social Anxiety				-0.38	-13.21***

Table S6: Testing the Mediation Model of Passive Usage and Negative Affect

Outcome Variable	Predictor Variable	R	R^2	F	β	t
Negative Affect		0.13	0.02	4.67***		
	ADUT				-0.07	-2.39*
	gender				0.07	2.23
	age				0.07	2.13
	Passive Usage				-0.04	-1.20
Social Anxiety		0.31	0.10	29.07***		
	ADUT				0.06	1.85
	gender				-0.23	-7.75***
	age				-0.13	-4.46***
	Passive Usage				0.13	4.42***
Negative Affect		0.40	0.16	40.44***		
	ADUT				0.05	1.82
	gender				0.02	0.74
	age				-0.01	-0.48
	Passive Usage				-0.02	-0.51
	Social Anxiety				0.40	13.43***
